# Medical Errors Cause Harm in Veterinary Hospitals

**DOI:** 10.3389/fvets.2019.00012

**Published:** 2019-02-05

**Authors:** Jessica Wallis, Daniel Fletcher, Adrienne Bentley, John Ludders

**Affiliations:** ^1^Department of Clinical Sciences, College of Veterinary Medicine, Cornell University, Ithaca, NY, United States; ^2^Cornell University Veterinary Specialists, Stamford, CT, United States

**Keywords:** medical error, incident, reporting, adverse, veterinary, safety, harm

## Abstract

Medical errors are a leading cause of mortality in human medicine. In contrast, errors in veterinary medicine are rarely discussed, and there is little known about their nature and frequency. This study aimed to evaluate the type and severity of medical errors reported in three veterinary hospitals. The voluntary online incident reporting systems of a small animal teaching hospital, large animal teaching hospital, and small animal multi-specialty practice were reviewed. Reports were included if they were entered between February 2015 and March 2018, and involved an incident pertaining to patient safety. The reporting systems classified errors into the following categories: drug, iatrogenic, system, communication, lab, oversight, staff, or equipment errors. In addition, all incidents were classified as resulting in either a near miss, harmless hit, adverse incident, or unsafe condition. Adverse incidents were further evaluated retrospectively for error severity. A total of 560 incident reports were included for analysis. Drug errors were the most frequently reported in all three hospitals, followed by failures of communication. Errors most commonly reached patients without causing harm (45%); however, 15% of all incidents resulted in patient harm. Eight percent of patients harmed suffered permanent morbidity or death. A higher proportion of adverse incidents were reported in the small animal teaching hospital than in the other two practice settings. This study demonstrates that medical errors have a substantial impact on veterinary patients. Establishing that drug and communication errors are most frequent in a variety of hospitals is the first step toward interventions to improve patient safety and outcomes in veterinary medicine.

## Introduction

Health care providers universally strive to provide a safe environment for their patients; however, human error is unavoidable. A medical error has been defined as “an act of omission or commission in planning or execution that contributes or could contribute to an unintended result” (1). Such incidents are well-recognized as a leading cause of morbidity and mortality in human hospitals, and recently have been estimated to be the third leading cause of death in the United States (2). Along with patient harm, errors may cause significant financial loses and have a negative impact on the well-being of the health care providers who feel responsible (3, 4). Despite the fact that human error is inevitable, systems can be developed to reduce their frequency and minimize their impact on patients (5, 6).

The first step in reducing errors is to identify them, whether or not they reach the patient or cause harm. By evaluating records of reported errors one can perform root cause analysis to identify contributing factors to the incident, and thus develop interventions to limit the occurrence of similar errors in the future (7). A number of different methods have been utilized to record errors; these include medical record review, direct observation of patient care, and voluntary incident reporting systems (6, 8–11).

A large number of studies investigating causes of medical error and approaches to reducing them have been published over the past two decades in the human medical field. Unlike in human medicine, there has not been an emphasis on developing a culture of safety and awareness of medical errors in veterinary medicine. There remains an unwillingness to admit to and discuss mistakes. Thus, there is a paucity of information in the veterinary literature. There are only a small number of veterinary publications that assess the impact of errors in veterinary medicine, with no large scale reports of hospital-wide error frequency (12–15). Even less attention has been paid to error reduction, with only a handful of reports examining specific aspects such as surgical errors or anesthetic mortality (16, 17). There are no reports of error reporting systems in veterinary medicine.

This study evaluates the results of an incident reporting system used in three veterinary practices: a university small animal hospital (SAU), university large animal hospital (LAU), and private practice referral/emergency small animal hospital (SAP). The purpose of the study was to determine the type and severity of errors in these practices. A secondary goal of this study was to evaluate for differences in the distribution of errors between teaching hospitals and private referral hospital. We hypothesized that drug errors would be the most frequently reported error type, and that adverse incidents causing patient harm would be more frequently reported in teaching hospitals than a private referral hospital.

## Materials and Methods

### Incident Reporting Survey Composition

A voluntary online incident reporting system was instituted in the SAU and LAU of the Cornell University Hospital for Animals in February of 2015 and slightly modified in August 2016. The same incident report was instituted in the SAP in January of 2016. These hospitals focus on emergency and referral cases, with primary care patients underrepresented. With the exception of the emergency services at the three hospitals, most clinicians work exclusively during weekday, daytime hours. After hours coverage is handled by a group of rotating clinicians who work a combination of day shifts, swing shifts, and overnight shifts. The nursing staff generally works consistently assigned shifts during either daytime, swing, or overnight hours. Pharmacy staff is only present in the hospital during daytime hours, and front desk staff work consistent day, weekend, or swing shifts. At the LAU and SAU students are primarily present during the day time hours although do occasionally have swing shifts in the emergency service. Each patient has an assigned student and that student will be responsible for some of the patient's in hospital treatments, as well assist in or perform procedures under direct supervision. Conversely, the SAP has only occasional student externs and so for the majority of patients procedures are performed exclusively by clinicians, and patient care is the responsibility of veterinary technicians.

Any person involved in a medical error was encouraged to fill out the voluntary online report form. The form was readily available online via a link on the hospital's internal home page. Reports could be filled out anonymously if preferred by the reporter. Immediately after submission the report was reviewed by an administrative staff member or veterinary technician assigned to this task and if additional information was required attempts were made to obtain it from the respondent or from the medical record.

When submitting an incident report, reporters chose from one or more incident types (Table 1) based on the DISCLOSE system used in human medicine (8). Prior to September 2016, in terms of the effect of an error on a patient, the reporter had three options to select from: patient safety event (reached the patient with or without harm), potential patient safety event (error did not reach patient), or unsafe conditions. After August 2016 the possible effects of an error on the patient was modified to more closely align with terms used in the medical error literature (Table 2).

**Table 1:** Error types and their definitions in the incident reporting system.

**Type**	**Definition**
Drug	Any issue with drug administration
Iatrogenic	Complication from procedure or treatment other than a drug
System	Delays, missed treatments, protocol issues
Communication	Misidentified patient, confusion over orders, failure to share information
Labs	Lost specimens, mislabeled samples, results not reported, delays, improper studies
Oversight	Judgment issues, missed diagnosis, misinterpretation of data, deviations from standard of care
Staff	Insufficient staff numbers, lack of access to needed staff, incident during staff training
Equipment	Inaccessibility, wrong equipment, failures, supply problems

**Table 2:** Incident types and their definitions.

**Incident type**	**Definition**
Near miss	Error did not reach the patient, but could have caused harm if it had
Harmless hit	Error reached the patient but did not cause harm
Adverse incident	Error reached the patient and caused harm
Unsafe condition	Circumstance or condition that increases the probability of a patient safety event

Additional questions sought the location of the incident and the occupation of the person submitting the report. A full description of the incident was also required. Additional information was requested for incidents involving drug and communication errors due to the importance of these errors as seen in human medicine and in order to collect information applicable only to these types of incidents. Drug errors were categorized as either wrong patient, wrong drug, wrong route, wrong time, or wrong dose. Communication errors were further defined as a failure of the source (missing information/incomplete information), failure of transmission (illegible handwriting, poor medium for transmitting information), or receiver failure (information forgotten or incorrectly interpreted) (18).

### Data Collection

Reports submitted between February 2015 to March 2018 for SAU and LAU, and between January 2016 to March 2018 for SAP, were included in the study if they involved a patient safety issue. Duplicate reports of the same incident in the same patient were excluded. Additional exclusion criteria included incomplete information such that error type and patient outcome could not be determined.

For reports submitted prior to September 2016, incidents defined as patient safety events were retrospectively categorized for all three hospitals as either adverse incidents or harmless hits by the authors. If additional information regarding the type of drug or communication error was not provided in the report, it was determined retrospectively by the authors if there was sufficient information. The severity of errors was retrospectively evaluated by the authors to determine if an adverse incident caused death, permanent harm, or temporary harm. The total number of patient visits to each hospital during the study period was determined.

### Statistical Analysis

Pearson's chi-squared tests were used to compare proportions of incident types between the three practice settings. When this test showed a significant difference in proportions, pair-wise chi-squared tests were used to identify significant differences in proportions between the individual practices. Significance was adjusted for multiple comparisons using the Bonferroni correction. All analyses were done using commercial statistical software (SPSS, IBM Corporation, Armonk, NY, USA).

## Results

A total of 651 incidents were reported during the study period. Ninety-one reports were excluded due to duplication, incomplete information, or not pertaining to patient safety. A total of 560 reports were therefore included for analysis. The total visits in each hospital during the reporting period and the number of incidents per 1,000 visits for each hospital were tabulated (see Table 3).

**Table 3:** Numbers of incident reports, patient visits, and errors per 1,000 patient visits for each hospital (see Figure 1 for hospital abbreviations).

**Hospital**	**Incident reports**	**Patient visits during study period**	**Errors/1,000 patient visits**
SAU	258	67,917	3.8
LAU	51	7,856	6.5
SAP	251	30,175	8.3
Total	560	105,948	5.3

**Figure 1:**
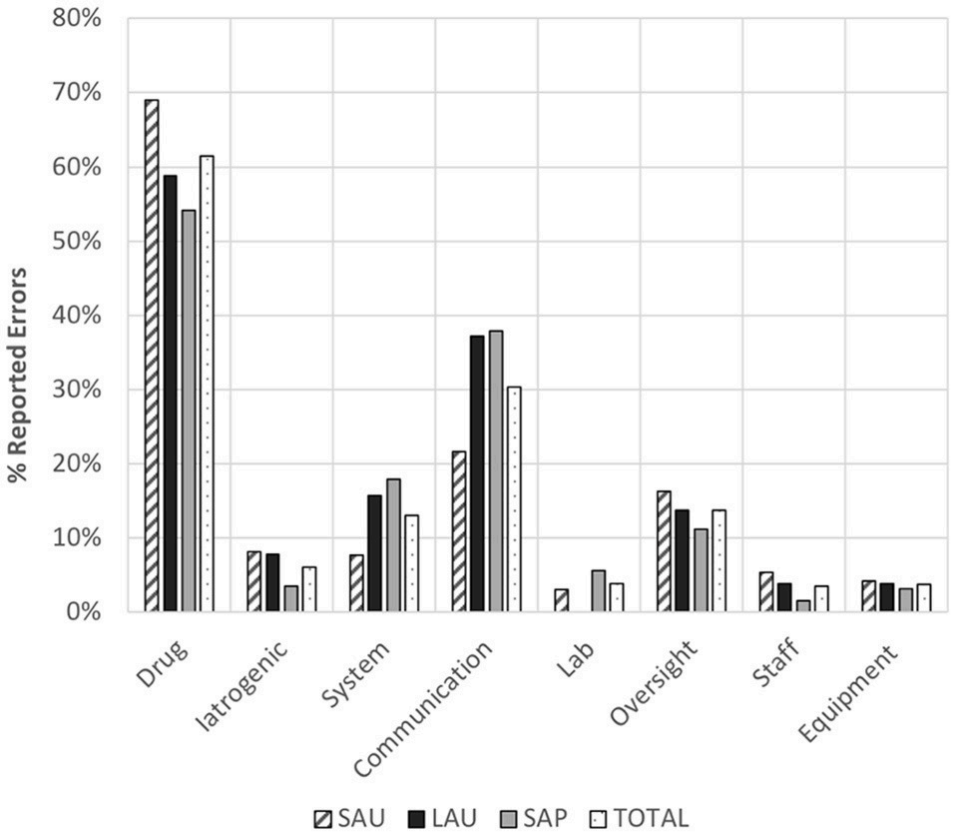
Distribution of proportion of error types with respect to the total number in incidents reported in each of the three veterinary hospitals (NB: totals for a given hospital may be >100% as multiple error types could be included in an incident report). SAU, small animal teaching hospital; LAU, large animal teaching hospital; SAP, small animal referral practice.

In all hospitals, drug errors were the most frequently reported safety incidents, followed by communication errors. Lab, staff, and equipment errors were rare (Figure 1). Because incidents for a hospital could involve multiple error types, percentages in the figure for a hospital may exceed 100%.

In this study, 45% of all errors reached patients but without causing harm; however, 15% resulted in patient harm (Table 4). Pearson's chi-square analysis showed a significant *(p*<*0.001)* difference in the distribution of error types across the three hospitals. Pairwise chi-square analysis with Bonferroni correction showed that a higher proportion of reported incidents were near misses in the SAP than in either the SAU or the LAU. A significantly higher proportion of reported incidents were adverse incidents in the two teaching hospitals compared to the SAP. In addition, a higher proportion of reported incidents involved unsafe conditions in the teaching hospitals than in the SAP.

**Table 4:** Distribution of incident types at each hospital (see Figure 1 for hospital abbreviations).

**Incident type**	**SAU number of errors (%)**	**LAU number of errors (%)**	**SAP number of errors (%)**	**Total number of errors (%)**
Near miss	31 (12.0)^a^	5 (9.8)^b^	71 (28.3)^a^, ^b^	107 (19.1)
Harmless hit	116 (45.0)	18 (35.3)	124 (49.4)	258 (46.1)
Adverse incident	51 (19.8)^c^	11 (21.6)^d^	23 (9.2)^c^, ^d^	85 (15.2)
Unsafe condition	60 (23.3)^e^	17 (33.3)^f^	33 (13.1)^e^, ^f^	110 (19.6)

*Like superscripts indicate significant difference*.

a, f *p ≤ 0.001*.

c, e *p ≤ 0.01*.

b, d *p < 0.05*.

In terms of patient outcome following an adverse incident, a majority (>82%) of patients had temporary harm (Table 5). A total of seven reports from all three hospitals reported permanent harm to a patient or death. These two patient outcomes are approximately 5% of all adverse incidents, three each in the SAU and LAU, and one in the SAP. Due to the small numbers, differences between the hospitals could not be determined statistically.

**Table 5:** Distribution of severity of patient harm due to adverse incidents at each hospital (see Figure 1 for hospital abbreviations).

**Severity of harm**	**SAU number of errors (%)**	**LAU number of errors (%)**	**SAP number of errors (%)**	**Total number of errors (%)**
Temporary harm	48 (94.1)	8 (82.7)	22 (95.7)	78 (91.8)
Permanent harm	0	2 (18.2)	1 (4.3)	3 (3.5)
Death	3 (5.9)	1 (9.1)	0 (0)	4 (4.7)

For all hospitals, drug errors accounted for the largest number of reported errors (>54%), and were most often due to the administration of the wrong dose (>40% in all hospitals). The proportions of each type of drug error differed significantly between hospitals *(p*<*0.001)*. In the LAU a higher proportion of reported errors were due to administration of the wrong drug than in the SAP. In the SAP, a higher proportion of reported drug errors were due to drug administration at the wrong time compared with the SAU *(p* = *0.001)* (Table 6).

**Table 6:** Distribution by type of drug error and hospital (see Figure 1 for hospital abbreviations).

**Drug errors**	**SAU number of errors (%)**	**LAU number of errors (%)**	**SAP number of errors (%)**	**Total number of errors (%)**
Wrong patient	6 (3.5)	0 (0)	7 (5.2)	13 (3.8)
Wrong drug	37 (21.4)	10 (34.5)^a^	15 (11.2)^a^	62 (18)
Wrong dose	111 (64.2)	13 (44.8)	75 (56)	199 (57.8)
Wrong route	7 (4.0)	4 (13.8)	9 (6.7)	20 (5.8)
Wrong time	12 (6.9)^b^	2 (6.9)	28 (20.9)^b^	42 (12.2)

*Like superscripts indicate significant difference*.

a *p = 0.005*.

b *p = 0.001*.

For communication errors there was no significant difference between the hospitals for the proportions of source, transmission or receiver errors *(p* = *0.2)*. These data are summarized in Table 7.

**Table 7:** Distribution by type of communication error for each hospital (see Figure 1 for hospital abbreviations).

**Communication errors**	**SAU number of errors (%)**	**LAU number of errors (%)**	**SAP number of errors (%)**	**Total number of errors (%)**
Source	20 (38.5)	6 (31.6)	33 (37.1)	59 (34.7)
Transmission	18 (34.6)	6 (31.6)	42 (47.2)	66 (38.8)
Receiver	14 (26.9)	7 (36.8)	14 (15.7)	35 (20.6)

There were significant *(p*<*0.001)* differences in the proportions of reporters in each occupation between hospitals. SAU reports were more often submitted by a technician than a doctor while in the LAU and SAP they were more often submitted by a doctor (Table 8).

**Table 8:** Distribution by occupation of reporters submitting incident reports at each hospital (see Figure 1 for hospital abbreviations).

**Occupation of reporters**	**SAU number of errors (%)**	**LAU number of errors (%)**	**SAP number of errors (%)**	**Total number of errors (%)**
Doctor	67 (36.7)^a^, ^b^	26 (53.1)^a^	102 (40.5)^b^	195 (35.3)
Technician	162 (64.5)^c, d^	13 (26.5)^c^	93 (36.9)^d^	268 (48.6)
Attendant/assistant/student	13 (5.2)	7 (14.3)^e^	8 (3.2)^e^	28 (5.1)
Non-technical staff	0 (0)	0 (0)	37 (14.7)	37 (6.7)
Pharmacist	9 (3.6)	3 (6.1)	5 (2)	17 (3.1)
Other	0 (0)	0 (0)	7 (2.8)	7 (1.3)

*Other refers primarily to administration, management and client service staff*.

*Like superscripts indicate significant difference*.

a, c, d *p ≤ 0.001*.

b, e *p = 0.003*.

## Discussion

This study demonstrates that medical errors occur frequently in veterinary hospitals, with approximately five errors reported per 1,000 patient visits across the three practice settings. The number of reports per patient visit was higher in the SAP but because of the nature of this study we are unable to draw conclusions as to the true prevalence of errors. Voluntary reporting systems underestimate the occurrence of errors, and the number reported may be more reflective of reporting practices of the hospital staff than the frequency of errors (9, 11, 19). Thus, rather than concluding that the SAP had an increased frequency of patient safety incidents, it seems most likely that hospital staff are simply more vigilant at reporting errors.

In most cases the incidents reported did not result in patient harm, which is a pattern similar to that seen with voluntary reporting in human hospitals (20). However, many near miss events have the potential to cause significant morbidity or mortality (9). Investigating all incidents, whether or not they result in harm, is important when developing interventions for error reduction (7). In this study the SAP had a lower frequency of adverse incidents than the SAU and LAU. This suggests that errors in veterinary teaching hospitals may be less likely to be detected prior to reaching the patient, and more likely to result in harm. It is the nature of teaching hospitals to have a large number of students, junior staff members, and new house officers. They may be less familiar with patient safety policies, and less experienced with many aspects of patient care and case management. Thus, medical errors may become a significant cause of patient harm in these settings. This has been demonstrated in human hospitals in the United States, with an increase in fatal errors seen with the intake of new medical residents in July (21). However, due to the voluntary nature of the survey, the smaller proportion of adverse incidents at the SAP may also be simply a reflection of increased staff reporting of less significant errors. This may be due to a higher proportion of staff at the SAP having more experience with the error reporting system than at the teaching hospitals, where new students and house officers are frequently entering the hospitals.

The frequency of serious errors causing permanent harm or death was low, at <2 percent of reported incidents. However, while they remain uncommon, such errors can have catastrophic consequences. It is important to recognize not only the harm to the patient, but also the emotional impact on the client, the damage to a hospital's reputation, and the mental health of those who feel responsible for the incident. Medical errors have been shown to contribute significantly to emotional burnout amongst healthcare providers, and veterinarians have reported a negative effect on their confidence and mental health after serious errors (3, 4, 14). Many of these errors can be avoided if appropriate systems are introduced to reduce risk. This highlights the importance of increasing awareness and research into medical errors in veterinary medicine, and the implementation of reporting systems and incident review committees to regularly evaluate the data from these systems. It is also vital that hospitals have systems in place that allow veterinarians to feel comfortable admitting mistakes and guide them through disclosing errors to clients. Human physicians have an obligation to disclose errors to their patients, and the way that an error is discussed has a significant impact on the patient's emotional response to the incident (22). The same is likely true for clients whose pets have been harmed by medical errors.

Drug errors were the most frequently reported error type in all three hospitals, accounting for between 55 and 69% of all errors. These findings are echoed by a previously published evaluation of errors in the anesthesia department of a veterinary teaching hospital, in which drug errors were also the most frequently reported (17). Drug errors at the SAP were more likely due to drug administration at the wrong time than at other hospitals. Each hospital had different policies and procedures for ordering, approving, and administering patient treatments, differences that likely influence the distribution of drug error types. This study was not designed to evaluate specific differences in how drugs were managed in the three hospitals and which may have contributed to the different types of drug errors reported across the three hospitals. In human hospitals medication errors are similarly frequent, and much research has been directed toward reducing adverse drug events (9, 23). In human hospitals, successful interventions include computerized physician order entry, dose error reduction systems, barcoded medication delivery systems, clinical pharmacists, and periodic but regular physician training. These protocols have been shown to reduce medication errors in a number of prospective trials and meta-analyses (7, 9, 24–27).

Communication errors were the second most commonly reported error type in all hospitals, which highlights the importance of encouraging effective case handover and team work in veterinary medicine. For information to be effectively communicated the first step involves a member of the team choosing when, where and how to communicate, and which information to present. This information is then transmitted to another team member via either electronic systems, hand written notes, treatment sheets, or verbal instruction. Finally, the receiver must then accurately hear, interpret, and remember the information. This final step is particularly susceptible to error in the case of handwritten notes, which may be difficult to read. If any step in this process fails information may be lost or miscommunicated, significantly impacting patient care. Our study demonstrated that regardless of hospital type each step of the communication process (source, transmission, and receiver) is equally important to ensure veterinary patient safety. In human medicine, failures of communication are well-recognized contributors to medical errors and adverse drug events (28, 29). Interventions targeted at improving staff dynamics and communication are an important component of improving patient care (30).

Compared to the other hospitals, a smaller proportion of incident reports in the SAU were submitted by doctors and a higher proportion were submitted by technicians. The pattern seen in the SAU mimics what is seen human hospitals in that physicians typically submit <5 percent of incident reports (20, 31, 32). The SAU hospital has a particularly high turnover of house officers due to residency and internship training programs. In contrast, many SAU technicians are highly trained and have many years of experience working in the hospital. It is also possible that the experienced staff have an increased awareness of medical errors, how they occur and why. That coupled with familiarity of the incident reporting system may explain why technicians more frequently submitted incident reports in the SAU as compared to the SAP. In the SAP, doctors and technicians likely have similar levels of experience with the system due to the small number of house officers in that setting and a low turnover in doctors. This staff dynamic may explain in part the differences identified as to whom submitted incident reports across the three hospitals, as the SAP has a higher proportion of experienced doctors directly involved in patient care. Alternatively, this difference may simply due to a difference in reporting culture, with the SAU doctors seeing error reporting as a task that technicians are responsible for. This suggests the need for more training of house officers in teaching hospitals to ensure that incidents are reported.

While our study demonstrates the important impact of medical errors in veterinary hospitals, it has significant limitations. Because of the voluntary nature of the incident reporting system, our results are prone to reporting bias. Staff may be more likely to report events associated with patient harm because they may perceive these incidents as more important than those that do not cause patient harm (20, 33). Conversely, serious adverse events may not be reported, or may be underreported due to concern on how such reports may impact an employee's career (34). The incident reporting system of this study allows for anonymous reports so as to reduce this concern. Overall, voluntary systems result in underreporting of errors (9, 11, 19). In studies of error rates in human hospitals voluntary reporting results in detection of as few as 3–5% of errors when compared to record review (35, 36). Factors that contribute to poor response rates include lack of familiarity with reporting systems, lack of time, fear of blame, and lack of motivation for reporting. These complicating factors have been described in more detail elsewhere (34, 37).

As a hospital's safety culture and awareness improve, the number of incidents reported is likely to increase (38). This may be misinterpreted as an increase in errors in the hospital despite a true decrease in incident frequency. These are important factors to consider as incident reporting systems are implemented and the safety culture of the hospital matures. Other options for error detection that have been shown to minimize this effect and avoid under-detection include direct observation and medical record review (10). Unfortunately, these systems may be less practical and affordable for long term implementation in veterinary hospitals.

Another limitation of our study is that most information was included exactly as reported by the person submitting the survey, but a small proportion was determined retrospectively by the authors. This was either due to incomplete data entry in anonymous reports, or due to the changes in the incident report in September 2016 as described above. Additionally, the severity of adverse events was retrospectively assigned. The retrospectively assigned data may have differed from what would have been entered by the reporter who originally submitted the report because at the time of incident, those involved may not have been aware of the true severity. While the staffing required to independently assess the severity of errors at the time the reports were submitted would be substantial, it would likely increase the accuracy of error severity reporting.

Finally, the distribution of medical errors reported here may not be reflective of all veterinary hospitals. By including both small animal and large animal as well as university and private practice hospitals, we attempted to provide a more global reflection of medical error reporting in the wider veterinary industry. However, the hospitals of this study have a focus on emergency and referral cases, with primary care patients underrepresented. We have demonstrated that there is a wide variation of error type, severity and reporting practices between individual hospitals. Thus, caution should be taken when applying these data to other institutions.

Further research in the field of veterinary medical errors is needed, and many questions remain unanswered by this study. These include the influence of the time of day on error frequency and severity, as well as the effects of illness severity and the number of treatments a patient is receiving. Future studies evaluating these factors may help determine which patients are at the highest risk of harm due to error.

This is the largest published evaluation of medical error type and severity in veterinary hospitals. We have demonstrated that errors can have a substantial impact on our patients and result in morbidity and mortality. Drug errors were the most frequently reported in three different hospital settings, but errors stemming from miscommunication were also common. Voluntary incident reporting systems are feasible for veterinary hospitals and yield useful information about the causes of errors. Future work is needed to determine if these systems may facilitate the development of interventions to improve safety and thus outcomes in veterinary patients.

## Datasets are Available on Request

The raw data supporting the conclusions of this manuscript will be made available by the authors, without undue reservation, to any qualified researcher.

## Author Contributions

DF, JL, and AB designed and implemented the incident reporting survey. JW, DF, and JL contributed to the design of the study. JW and JL performed data collection and retrospective review of data. DF performed the statistical analysis. JW wrote the first draft of the manuscript. All authors contributed to manuscript revision.

### Conflict of Interest Statement

The authors declare that the research was conducted in the absence of any commercial or financial relationships that could be construed as a potential conflict of interest.

## Abbreviations

SAU: small animal university hospital
LAU: large animal university hospital
SAP: private practice referral/emergency small animal hospital.

## References

[1] Grober ED Bohnen JMA. Defining medical error. Can J Surg. (2005) 48:39–44. 15757035 PMC3211566

[2] Makary MA Daniel M. Medical error—the third leading cause of death in the US. BMJ (2016) 353:i2139. 10.1136/bmj.i2139 27143499

[3] West CP Huschka MM Novotny PJ Sloan JA Kolars JC Habermann TM. Association of perceived medical errors with resident distress and empathy. JAMA (2006) 296:1071. 10.1001/jama.296.9.1071 16954486

[4] Shanafelt TD Balch CM Bechamps G Russell T Dyrbye L Satele D. Burnout and medical errors among american surgeons. Ann Surg. (2010) 251:995–1000. 10.1097/SLA.0b013e3181bfdab3 19934755

[5] Kohn LT Corrigan JM Donaldson MS To err is human: building a safer health system. In: Kohn LT Corrigan JM Donaldson MS, editors. Institute of Medicine (US) Committee on Quality of Health Care in America. Washington, DC: National Academy Press (2000) 51–66. 25077248

[6] Garrouste-Orgeas M Philippart F Bruel C Max A Lau N Misset B. Overview of medical errors and adverse events. Ann Intensive Care (2012) 2:2–10. 10.1186/2110-5820-2-2 22339769 PMC3310841

[7] Pham JC Aswani MS Rosen M Lee H Huddle M Weeks K. Reducing medical errors and adverse events. Annu Rev Med. (2012) 63:447–63. 10.1146/annurev-med-061410-121352 22053736

[8] King ES Moyer D V, Couturie MJ, Gaughan JP, Shulkin DJ. Getting doctors to report medical errors: project DISCLOSE. Jt Comm J Qual Patient Saf. (2006) 32:382–92. 10.1016/S1553-7250(06)32050-8 16884125

[9] Rothschild JM Landrigan CP Cronin JW Kaushal R Lockley SW Burdick E. The critical care safety study: the incidence and nature of adverse events and serious medical errors in intensive care. Crit Care Med. (2005) 33:1694–700. 10.1097/01.CCM.0000171609.91035.BD 16096443

[10] Leape LL. A systems analysis approach to medical error. J Eval Clin Pract. (1997) 3:213–22. 10.1046/j.1365-2753.1997.00006.x 9406109

[11] Murff HJ Patel VL Hripcsak G Bates DW. Detecting adverse events for patient safety research: a review of current methodologies. J Biomed Inform. (2003) 36:131–43. 10.1016/J.JBI.2003.08.003 14552854

[12] Kogan LR Rishniw M Hellyer PW Schoenfeld-Tacher RM. Veterinarians' experiences with near misses and adverse events. J Am Vet Med Assoc. (2018) 252:586–95. 10.2460/javma.252.5.586 29461160

[13] Oxtoby C Ferguson E White K Mossop L. We need to talk about error: causes and types of error in veterinary practice. Vet Rec. (2015) 177:438. 10.1136/vr.103331 26489997

[14] Mellanby RJ Herrtage ME. Survey of mistakes made by recent veterinary graduates. Vet Rec. (2004) 155:761–5. 10.1136/VR.155.24.761 15637999

[15] Kinnison T Guile D May SA. Errors in veterinary practice: preliminary lessons for building better veterinary teams. Vet Rec. (2015) 177:492. 10.1136/vr.103327 26494771

[16] Bergström A Dimopoulou M Eldh M. Reduction of surgical complications in dogs and cats by the use of a surgical safety checklist. (2016) 45:571–6. 10.1111/vsu.12482 27195524

[17] Hofmeister EH Quandt J Braun C Shepard M. Development, implementation and impact of simple patient safety interventions in a university teaching hospital. Vet Anaesth Analg. (2014) 41:243–8. 10.1111/vaa.12124 24571418

[18] Lingard L Espin S Whyte S Regehr G Baker GR Reznick R. Communication failures in the operating room: an observational classification of recurrent types and effects. Qual Saf Heal Care (2004) 13:330–4. 10.1136/qhc.13.5.330 15465935 PMC1743897

[19] Hamilton EC Pham DH Minzenmayer AN Austin MT Lally KP Tsao K. Are we missing the near misses in the OR?—underreporting of safety incidents in pediatric surgery. J Surg Res. (2018) 221:336–42. 10.1016/J.JSS.2017.08.005 29229148

[20] Milch CE Salem DN Pauker SG Lundquist TG Kumar S Chen J. Voluntary electronic reporting of medical errors and adverse events. J Gen Intern Med. (2006) 21:165–70. 10.1111/j.1525-1497.2006.00322.x 16390502 PMC1484668

[21] Phillips DP Barker GEC. A july spike in fatal medication errors: a possible effect of new medical residents. J Gen Intern Med. (2010) 25:774–9. 10.1007/s11606-010-1356-3 20512532 PMC2896592

[22] Gallagher TH Waterman AD Ebers AG Fraser VJ Levinson W. Patients' and physicians' attitudes regarding the disclosure of medical errors. JAMA (2003) 289:1001. 10.1001/jama.289.8.1001 12597752

[23] Miller MR Robinson KA Lubomski LH Rinke ML Pronovost PJ. Medication errors in paediatric care: a systematic review of epidemiology and an evaluation of evidence supporting reduction strategy recommendations. Qual Saf Heal Care (2007) 16:116–26. 10.1136/qshc.2006.019950 17403758 PMC2653149

[24] Nuckols TK Smith-Spangler C Morton SC Asch SM Patel VM Anderson LJ. The effectiveness of computerized order entry at reducing preventable adverse drug events and medication errors in hospital settings: a systematic review and meta-analysis. Syst Rev. (2014) 3:56. 10.1186/2046-4053-3-56 24894078 PMC4096499

[25] Wang T Benedict N Olsen KM Luan R Zhu X Zhou N. Effect of critical care pharmacist's intervention on medication errors: a systematic review and meta-analysis of observational studies. J Crit Care (2015) 30:1101–6. 10.1016/J.JCRC.2015.06.018 26260916

[26] Truitt E Thompson R Blazey-Martin D NiSai D Salem D. Effect of the implementation of barcode technology and an electronic medication administration record on adverse drug events. Hosp Pharm. (2016) 51:474–83. 10.1310/hpj5106-474 27354749 PMC4911988

[27] Ohashi K Dalleur O Dykes PC Bates DW. Benefits and risks of using smart pumps to reduce medication error rates: a systematic review. Drug Saf. (2014) 37:1011–20. 10.1007/s40264-014-0232-1 25294653

[28] Sutcliffe K Lewton E Rosenthal M. Communication failures: an insidious contributor to medical mishaps. Acad Med. (2004) 79:186–94. 10.1097/00001888-200402000-00019 14744724

[29] Leonard M Graham S. The human factor: the critical importance of effective teamwork and communication in providing safe care. Qual Saf Heal Care (2004) 13:85–90. 10.1136/qshc.2004.010033 15465961 PMC1765783

[30] Pronovost P Berenholtz S Dorman T Lipsett PA Simmonds T Haraden C. Improving communication in the ICU using daily goals. J Crit Care (2003) 18:71–5. 10.1053/JCRC.2003.50008 12800116

[31] Rowin EJ Lucier D Pauker SG Kumar S Chen J Salem DN. Does error and adverse event reporting by physicians and nurses differ? Jt Comm J Qual patient Saf. (2008) 34:537–45. 10.1016/S1553-7250(08)34068-9 18792658

[32] Tuttle D Holloway R Baird T Sheehan B Skelton WK. Electronic reporting to improve patient safety. Qual Saf Heal Care (2004) 13:281–6. 10.1136/qhc.13.4.281 15289631 PMC1743869

[33] Osmon S Harris CB Dunagan WC Prentice D Fraser VJ Kollef MH. Reporting of medical errors: an intensive care unit experience. Crit Care Med. (2004) 32:727–33. 10.1097/01.CCM.0000114822.36890.7C 15090954

[34] Uribe CL Schweikhart SB Pathak DS Dow M Marsh GB. Perceived barriers to medical-error reporting: an exploratory investigation. J Healthc Manag. (2002) 47:263–81. 10.1097/00115514-200207000-00009 12221747

[35] Hanskamp-Sebregts M Zegers M Vincent C van Gurp PJ de Vet HCW Wollersheim H. Measurement of patient safety: a systematic review of the reliability and validity of adverse event detection with record review. BMJ Open (2016) 6:e011078. 10.1136/bmjopen-2016-011078 27550650 PMC5013509

[36] Classen DC Resar R Griffin F Federico F Frankel T Kimmel N “Global trigger tool” shows that adverse events in hospitals may be ten times greater than previously measured. Health Aff. (2011) 30:581–9. 10.1377/hlthaff.2011.0190 21471476

[37] Evans SM Berry JG Smith BJ Esterman A Selim P O'shaughnessy J. Attitudes and barriers to incident reporting: a collaborative hospital study. Qual Saf Heal Care (2006) 15:39–43. 10.1136/qshc.2004.012559 16456208 PMC2563993

[38] Hutchinson A Young TA Cooper KL McIntosh A Karnon JD Scobie S. Trends in healthcare incident reporting and relationship to safety and quality data in acute hospitals: results from the National Reporting and Learning System. Qual Saf Health Care (2009) 18:5–10. 10.1136/qshc.2007.022400 19204125

